# Supplementary material for: Are carbon emissions trading and green financial instruments synergistic? -Comprehensive quantitative research based on content analysis
Source: PLoS One. 2024 Mar 7;19(3):e0298601. doi: 10.1371/journal.pone.0298601 (PMC10919601; doi:10.1371/journal.pone.0298601)
Supplement: S1 Data — (ZIP) [file pone.0298601.s001.zip › Data/地级市Z_F工作报告.zip!/║■─╧/│ú╡┬╩╨/└┤╫╘╣1⁄2╓┌║┼í╛┬φ┐╦╩2╛▌═°í┐_2015.docx]

各位代表：

现在，我代表市人民政府，向大会报告政府工作，请予审查，请各位政协委员和其他列席人员提出意见。

过去一年

坚持出实招，奋发干实事，努力求实效

2014年，面对前所未有的困难和挑战，我们在市委的坚强领导下，和全市人民一道，务实创新，砥砺前行，着力打好民生升温、园区攻坚、城市提质三大战役，扎实推进新常德新创业，干成了一批大事，办好了一批实事，攻克了一批难事，实现了经济社会持续健康发展。回顾过去一年，我们深感不平凡、不容易、不简单。

一、奋力稳增长进一步巩固在省内的位置，发展目标较好实现。坚持把稳增长、促发展作为头等大事，克服宏观经济下行压力，及时研判，多措并举，实现了经济发展稳中有进、稳中向好，在全省靠前的位置更加巩固。全年完成地区生产总值2520亿元，增长10%(预计数，下同)；固定资产投资1600亿元，增长25%；财政总收入185亿元，增长10%；城镇居民人均可支配收入22842元，增长10%；农民人均可支配收入10726元，增长11.4%。

突出投资拉动。以项目扩投资、以投资稳增长，全年产业建设、基础设施、民生项目投资分别增长20.7%、36.9%、48.7%。年初确定的500个重点建设项目，完成投资829亿元，其中237个项目竣工投产；500个重大前期项目，有168个项目提前开工建设。

拓展消费需求。完成社会消费品零售总额820亿元，增长13%。旅游消费扩大，全年旅游综合收入增长20%，梦幻桃花岛水公园完成主体工程，世外桃源古镇、江湖连通等精品工程加快推进，市规划展示馆、鼎城花岩溪成功创建国家4A级旅游景区。房地产消费总体平稳，电子商务、信息消费快速增长，养老、健康、文化等消费持续扩大。城市商圈、大型专业市场、物流园区建设提质提速，湘西北汽贸城、东星家居广场建设进展顺利。

加强要素保障。重视做好资金融通工作，争取上级投资和项目资金230亿元；金融机构新增各类贷款152亿元；市本级新增城建融资230亿元；新设立金融机构17家；惠生国际在港交所上市。优化用地和征拆服务，共批回建设用地2.3万亩，较好地保障了重点项目建设需求。强化科技和人才支撑，实施省级以上科技项目111项、产学研合作项目56个，新增院士工作站5家，全年专利授权量1109件；全面启动“四个一”人才工程建设，为企业输送高端人才176名。

二、引进和建设重大产业项目取得可喜进展，园区攻坚成效显著。打好园区攻坚战役，全市完成规模工业增加值942亿元，增长10%。

园区发展迈上新台阶。园区新一轮扩规修编基本完成，中联重科千亿工业走廊规划、常德经开区千亿园区规划、芙蓉王现代新城规划抓紧编制。市县共投入60亿元，实施园区道路、管网等基础建设项目262个，开工建设标准化厂房200万平方米，建成110万平方米 侯卫东官场笔记。全市园区新开工亿元项目61个，竣工投产亿元项目25个。

产业建设取得新进展。加快产业转型升级步伐，推动江北城区企业“退二进三”，淘汰落后产能，全年完成技改投入396亿元，新增中国驰名商标4件。落实“1115”工程企业“三特”政策，支持骨干企业发展壮大，芙蓉王品牌销售收入突破千亿大关。积极解决中小微企业在融资、用工、环境等方面的困难和问题，帮助企业渡难关、闯市场。全年新增规模工业企业77家。

重大项目实现新突破。全球知名的清洁能源公司汉能集团、国内信息技术领军企业浪潮集团、中国文化旅游第一品牌华侨城集团、国内著名商业地产企业万达集团、新能源汽车银泰集团等一批战略投资者成功引进；投资300亿元的忠旺铝材以及中国中车、中联现代农装等重大项目取得实质性进展；常德电厂一期、常德烟厂易地技扩改等重大项目建设扎实推进；恒安纸业扩建、雨润食品屠宰、常德烟机技改、美华尼龙扩建等项目建成投产。全年引进内外资总额551亿元，增长15.2%。

三、抓住全省推进新型城镇化工作会议在我市召开的契机，城市提质再谱新篇。以超常的谋划、超常的组织，打好城市提质战役，城市面貌日新月异，省委省政府向全省推介了常德推进新型城镇化的工作经验。

城市品质大幅提升。加强城市规划设计，基本完成高速公路环线内500平方公里总体城市设计，优化了城区空间发展布局。加快重点工程建设，建成太阳山通道，白鹤山、芦荻山和机场通道即将通车，桥南人防工程、柳叶湖环湖大道等项目进展顺利。“三改四化”深入推进，路改年度任务全面完成，武陵桥、朗州桥、七里桥、柏园桥4座城市景观桥梁建成通车；水改强力攻坚，穿紫河、新河渠、护城河改造工程加紧施工；棚改稳步实施，改造城市棚户区10419户；绿化、美化、亮化、数字化加快推进，丁玲公园建成开园，武陵阁步行城美化提质抓紧建设，城市亮化全面铺开，云计算中心、智能交通、智慧城管、数字防控、地理空间框架系统建成运行。全面开展交通秩序、市容市貌两项整治，城市卫生保洁水平有新提高。中心城区全年完成城建投入110亿元。

城镇建设整体推进。各县(市)城区“三改四化”和城市创建广泛开展，“一主一副四轴”城镇发展框架逐步形成。津澧新城建设进入洞庭湖生态经济区规划和国家中小城市综合改革试点，石门县澧阳大道、汉寿县龙阳大道提质改造基本完成，桃源县文化体育中心加快建设。临澧县新安镇、鼎城区蒿子港镇等17个镇成为全国重点镇，安乡县黄山头镇成为省际边界重点扶持镇。

完美社区加速建设。各级财政投入2.8亿元，新建改建街道“一所一校四中心”、社区“一站一园一场五室”项目193个。推进武陵区、鼎城区、常德经开区、柳叶湖旅游度假区、澧县的“改镇设办”工作，完成了部分社区区划调整。大力推行“3+N”治理模式，社区网格化管理工作获得中央综治委肯定，全省社区党建和社区建设工作会议以武陵区为现场，推介了我市完美社区建设经验。

四、解决城乡居民饮水安全工作走在全省前列，民生升温力度空前。打好民生升温战役，提升群众幸福指数。全年用于民生的财政支出达到262亿元，超过财政总支出的75%。省为民办实事28项指标全面达标，其中16项超过省定标准。

饮水安全问题加快解决。积极回应人民群众的期待，把解决城乡居民饮水安全问题作为幸福常德的一号工程来抓，全年投入资金7.7亿元，建设饮水工程281处，解决了151万人的饮水安全问题，相当于过去五年的总和。全省农村饮水安全工作推进会、长江流域农村饮水安全现场观摩会在我市召开。

就业和社会保障得到加强。全年新增城镇就业6.9万人，农村劳动力转移就业8.5万人。五类社会保险新增参保12万人次，发放社会保障资金65.3亿元。企业退休人员基本养老金月人均增加182元，农村低保扩面4.8万人，为全市5.7万名重度残疾人发放了护理补贴。开工建设保障性安居工程5.5万户。改扩建农村敬老院16所，建成了一批全省一流的示范养老机构。

教育卫生三年攻坚取得实效。教育方面：共改造农村薄弱学校146所，新建、改扩建城区中小学34所，新建城区幼儿园44所。湖南应用技术学院实现“专升本”，常德智慧谷和湖南幼师高专、常德技师学院新校区加快建设。开展了首届“翦伯赞教育突出贡献奖”的评选和表彰。高考成绩文科居全省第一，理科居全省第二。卫生方面：实施市县医疗卫生建设项目25个、城市社区卫生服务中心项目10个，市第一人民医院新住院大楼竣工使用，湘雅常德医院、石门县人民医院、临澧县人民医院建设等一批项目进展顺利。医药卫生体制改革进一步深化，城乡居民大病保险试点全面实施，基本医疗保障水平继续提高。重大传染病及突发公共卫生事件防控处置有力。

扶贫攻坚有力推进。开展产业扶贫、定点扶贫、精准扶贫，全市共投入2.4亿元，对300个重点贫困村实施“六件实事”扶贫攻坚行动，贫困村生产生活条件明显改善，年内有11万人脱贫。

各项社会事业协调发展。公共文化服务水平提高，丁玲纪念馆建成开馆，城头山博物馆完成主体工程，“百团大赛”、鼓书交流会等文化活动精彩纷呈。“扫黄打非”工作成效显著。全民健身运动广泛开展，参加省第十二届运动会取得了第二名的好成绩。计划生育基层基础进一步夯实，我市被评为全省人口和计划生育工作模范市。第三次全国经济普查稳步推开。第九次村委会换届选举顺利完成。审计、粮食、外事侨务、气象、防震减灾、档案、民族宗教、对台、方志、科协、贸促会、红十字会等工作取得新成绩。工会、共青团、妇联的作用进一步发挥。

国防教育、国防动员、人防建设、民兵预备役建设得到加强，“双带双促”、“双拥”共建活动深入开展，我市连续两届被评为省级“双拥”模范城。驻常人民解放军、武警部队和民兵预备役官兵为抗洪抢险和全市经济社会发展作出了重要贡献。

社会大局安全稳定。大力开展平安常德建设，严厉打击各类违法犯罪活动，完善社会治安防控体系，公众安全感得到提升。深入开展安全生产大整治，加强打非治违和隐患排查，没有发生较大以上安全生产事故，安全生产工作进入全省先进行列。食品药品专项整治不断加强，食用油放心工程扎实推进。畅通信访渠道，加强领导接访和下访包案，推进事要解决，治理违法信访，实施“三调联动”，一批信访积案和矛盾纠纷得到有效化解。

五、抗击沅水流域超历史的洪水取得决定性胜利，“三农”工作持续加强

抗洪抢险取得重大胜利。7月中旬，我市沅水流域遭遇了历史最高水位的洪水灾害，发生各类险情1000多处，其中重大险情10多处。面对严峻的险情灾情，省市防指科学调度，各级领导靠前指挥，广大军民奋力抢险，取得了不溃一堤一垸、不垮一库一坝、没有因灾死亡一人的好效果。

现代农业不断发展。粮食、肉类、水产、蔬菜等大宗农产品产量稳定增长。西湖西洞庭国家现代农业示范区建设加快推进，开展了100个小型园区建设，国家农业科技园创建工作全面启动。农业标准化、农业机械化、农业产业化建设加强，新增国家地理标志保护产品7个，新增省级以上农产品加工龙头企业12家。

美丽乡村加快建设。整合各类资金6.8亿元，大力推进“百村示范、千村创建、村村整治”行动，涌现了石门秀坪、柳叶湖太阳谷、桃源枫树、安乡出口洲等一批示范亮点。大力开展农村环境集中整治，禁止投肥养殖范围进一步扩大。

农村发展活力增强。全市新增农民合作社760家、家庭农场142家；流转耕地面积达到130万亩，占耕地总面积的20%；农村土地承包经营权确权登记颁证试点工作基本完成，农村土地经营权抵押贷款试点“汉寿模式”得到国家推介；新型职业农民培育整市推进试点工作扎实开展。

六、主攻道路交通建设发展瓶颈，基础设施加快改善

交通建设攻坚提速。黔张常铁路开工建设，石长铁路增建二线及电气化工程抓紧施工。桃花源机场扩建顺利推进。东常高速建成通车，益常高速大修工程竣工。新建改建干线公路165公里，周家店至西湖、三岔路至临澧、澧县县城至卷桥公路和澧水艳洲大桥建成通车，石龟山大桥重建、国道319和207市城区改线等项目开工。建设农村公路529公里，实施病危桥改造112座。盐关码头国家二类口岸竣工运行。公路治超取得较好成效。

水利建设势头强劲。全市投入水利建设资金40亿元，是建市以来投入最多、规模最大的。加固堤防207处，整治病险水库88座，治理中小河流16条，更新改造大型泵站6处。实施小型农田水利建设五年行动，扩容堰塘5.1万口，疏浚渠道2万公里。建成高标准农田和改造中低产田15.6万亩。全省水利建设现场会在我市召开。

能源建设步伐加快。桃源水电站全部机组投产发电。建成了一批大型输变电工程，启动了西湖、西洞庭、桃源县地方电网管理体制改革，完成了314个行政村的农网升级改造。建设“五县一市”天然气输配管道142公里。

“两型”建设持续加强。实施重点节能项目11个、重点减排项目82个，完成了节能减排约束性指标。开展大气污染治理，市城区空气质量达标率85.2%，比上年提升10.8个百分点。加强水环境综合治理，划定饮用水源地保护区267处，沅澧两水水质保持Ⅲ类以上标准。石门县纳入国家生态主体功能区建设试点，桃源县成为武陵山片区国家生态文明先行示范区，汉寿县、澧县、安乡县成为洞庭湖生态经济区生态补偿试点县。新造林28.7万亩，国家森林城市创建扎实开展。

七、突破城管体制下放等改革难点，各项改革深入推进。以敢于啃硬骨头、敢于涉险滩的精神抓改革，省委、省政府部署的48项改革稳步推进，市里安排的10项改革顺利实施，承接中央和省里的6项改革试点工作初见成效。

推进城管体制改革。将18项职权事权、13个管理机构和各类人员3000多名下放到区一级，较好地理顺了城市管理的体制机制，城市管理“一改四化”的目标正在逐步实现。

推进园区管理体制改革。市委、市政府出台“一号文件”，下放经济社会管理权限69项，支持常德经开区推进二次创业、打造千亿园区。各区县(市)园区“一权两制一司”管理模式基本建立。

推进投融资体制改革。进一步完善了市一级投融资公司法人治理结构，构建债务风险管控机制，支持企业开发经营性项目，投融资公司市场化转型步伐加快。

推进政府机构改革。市县政府转变职能和机构改革启动。公路体制改革基本完成，财税体制改革、事业单位分类改革、国有资产管理改革等取得积极进展。

八、践行群众路线厉行正风肃纪，政府效能明显提升

大力改进政风。扎实开展党的群众路线教育实践活动，形式主义、官僚主义、享乐主义和奢靡之风得到有效遏制。认真落实中央八项规定、省委九项规定和市委十项规定，开展公车私用、超标办公用房等专项治理，加强审计监督和行政监察，狠抓廉政建设，一批违法违纪案件得到查处，会议、文件、考核检查评比、奖励项目、“三公”经费等大幅减少，干部作风和社会风气明显好转。

优化发展环境。行政审批制度改革继续深化，取消、调整、下放、合并行政审批事项50项。开展部门涉企收费专项检查，治理部门办事“三难”问题，实施优化项目建设环境“五百”行动，严厉打击强行阻工、强揽工程、欺行霸市行为，全市查处相关案件82起，拘留74人。

严格依法行政。深入贯彻依法治国基本方略，建立健全科学民主决策机制，认真执行市人大及其常委会的决议决定，自觉接受市人大及其常委会的法律监督和工作监督，自觉接受市政协的民主监督，认真采纳政治协商成果，主动听取各民主党派、工商联、无党派人士的意见和建议。全年办理人大代表建议243件，办理政协提案513件。自觉接受社会监督和舆论监督，网络在线问政、市长热线、政府网站等互动平台进一步畅通。

各位代表!过去一年的发展可圈可点，过去一年的工作可敬可佩。这些成绩归功于市委的正确领导，归功于市人大、市政协的监督支持，归功于全市人民的团结奋斗，归功于政通人和、万众一心凝聚起来的强大常德力量。在此，我代表市人民政府，向辛勤工作在各条战线、各个岗位上的全市人民，向各位人大代表和政协委员，向各民主党派、工商联和社会各界人士，向驻常人民解放军、武警消防官兵和公安政法干警，向所有关心、支持常德发展的同志们、朋友们，表示衷心的感谢!

在充分肯定成绩的同时，我们也清醒地看到，当前经济社会发展和政府工作中还存在许多问题和不足：一是部分指标没有达到预期，受宏观经济形势严峻、市场需求疲软、投资扩张动能不足等因素影响，部分经济指标增速没有达到年初预期。二是重大项目储备不多，一些地方招商引资的效果不太明显，新的增长点还没有真正形成，经济转型发展、结构调整优化的任务还相当繁重。三是县域经济普遍不强，民营经济发展不够，经济外向度比较低，已经成为我们发展中亟待解决的“短板”。四是解决就业、就学、就医问题的困难依然较多，扶贫帮困、扶弱助残等救助水平还不高，维护民生民利的压力还很大。五是控制和治理污染、保护生态环境的工作与人民群众的期待还有落差。六是影响安全稳定的隐患和风险仍然存在，社会治理任务仍然艰巨，不可掉以轻心。七是干部队伍的思想观念、行为方式、工作作风还有很大的改进空间，还不同程度存在“伪现代”、“假开放”、“浅作为”等不良现象，存在作风不优、为官不为、为政不廉等消极腐败现象。这些都需要我们在今后的发展和工作中认真加以解决。

新的一年

适应新常态，抢抓新机遇，推动新发展

2015年，是全面深化改革和全面建成小康社会的关键之年，是全面推进依法治国的开局之年，也是全面完成“十二五”规划的收官之年。当前，经济社会发展进入了新阶段、呈现出新常态。

我们必须看到，新常态对我们提出了新要求。中央基于“三期”叠加的阶段性特征，作出了我国经济发展进入新常态的重大战略判断。在新常态下，增长速度、发展方式、经济结构、发展动力正在发生新的调整和转换。新常态，实质上是可持续发展的常态，是转方式调结构的常态，是创新驱动的常态，是绿色环保的常态，是重视民生民利的常态，是安全稳定的常态。认识新常态，适应新常态，引领新常态，是当前和今后一个时期经济发展的大逻辑。在这一历史进程中，常德作为后发地区，要特别注重处理好共性和个性、挑战和机遇、普遍要求和特殊作为的关系，既要顺应和适应大势，更要从实际出发，坚持发展，主动作为。

我们更应该看到，新常态给我们带来了新机遇。新常态正是我们实现“弯道超越”的有利时机。我们要把握好宏观政策持续稳定的机遇，中央继续实施积极的财政政策和稳健的货币政策，实施定向调控、结构性调控的措施，保持宏观经济运行在合理区间，推行以基础设施和民生项目为主体的补短板、稳投资政策，重点加强中西部铁路、水利、能源、生态环保等建设，为我们争投资、稳增长提供了强大支撑。我们要把握好区域发展“和而不同”的机遇，新常态下，发达地区主动调结构、促转型的力度会更大，我们作为欠发达地区，承接产业转移、实现赶超发展的机遇会更多。我们要把握好国家战略深入实施的机遇，国家重点实施“一带一路”、京津冀协同发展、长江经济带三大战略，为我们在更高层面和更多方位谋划发展、寻求合作带来了大好机遇，特别是我们作为长江经济带的重要节点城市，作为洞庭湖生态经济区的重要组成部分，更是身处其中、直接受益，必须超前谋划，主动融入，积极对接。我们要把握好体制改革持续发力的机遇，中央和省里全面深化改革，大力简政放权，激发内生动力，为地方赋予了更大的作为空间，为市场主体创新创业搭建了更加广阔的发展舞台，我们必须把改革的红利用好用活用足。

认识新常态，就要抢抓新机遇；适应新常态，就要善于新作为；引领新常态，就要敢于新突破。我们要努力增创新优势，推动新发展。

今年政府工作的总体要求是：全面贯彻落实党的十八大、十八届三中、四中全会以及中央、省委、市委经济工作会议精神，坚持稳中求进工作总基调，坚持新常德新创业总要求，主动适应经济发展新常态，以全面建成小康社会为总揽，更加突出改革攻坚，更加突出依法治市，更加突出创新驱动，更加突出风险防控，深入打好民生升温、园区攻坚、城市提质三大战役，努力保持经济平稳健康发展、社会和谐稳定。

经济社会发展的主要预期目标是：地区生产总值增长9.5%左右，规模工业增加值增长10%左右，固定资产投资增长20%左右，财政收入增长10%左右，城镇新增就业6万人以上，城镇居民和农民人均可支配收入增长10%左右，完成省里下达的节能减排目标任务。

新的一年，我们要举全市之力，推动一批具有带动性、支撑性、普惠性的重大建设 大主宰小说。①完成石长铁路增建二线及电气化改造，力争开通动车；②全面开工建设安慈高速公路；③完成桃花源机场4D级扩建并投入营运；④开工建设沅澧快速干线第一大道；⑤全面建设芙蓉王现代新城暨烟厂易地技扩改项目；⑥实现常德电厂一期工程投产发电；⑦全面开工建设以万达广场为标志的白马湖商务中心；⑧实现华侨城梦幻桃花岛水公园“6·1”盛大开园；⑨规划并启动建设柳叶湖现代服务业园区，完成湘雅常德医院主体工程；⑩全面解决剩余136万人的饮水安全问题，在全省率先实现城乡居民饮水安全全覆盖。

政府工作主要抓好以下五个方面：

一、突出发展主线，全力以赴稳增长

紧紧扭住产业发展这条主线，努力实现稳增长的目标。

大力发展新型工业。始终把新型工业化作为经济社会发展的第一推动力，继续实施园区攻坚，加快建设工业强市。

打好园区攻坚战役。推动园区建设发展转型，使工业园区成为主导产业、配套服务、高端人才、孵化创新、生态环保的高端集成。继续大力建设标准化厂房，提高标准化厂房利用率，全年新建标准化厂房150万平方米，完成园区基础建设投入50亿元以上。抓好园区体制机制创新，推动“一权两制一司”运行到位，增强园区发展活力。促进园区整合升级，积极推进以鼎城高新区为主体、以汉寿高新区、西洞庭生物科技园为拓展的国家级高新区创建；整合澧县、津市工业园区申报国家级经开区。对园区发展的考核，要有新增规模企业数量、引进重大项目等量化指标。

深入推进“1115”工程。加快建设千亿工业走廊，争取中联现代农装等项目早日落户。积极推进常德经开区二次创业、建设千亿园区，加快汉能光伏、武陵酒酱酒工业园等重大项目建设，全力以赴引进忠旺铝材、中国中车等战略投资者，全年技工贸总收入突破600亿元。夯实千亿烟草产业基础。认真落实“三特”政策，建立“1115”工程企业优选机制，甄选补充一批潜力大、增长快、效益好的企业进行重点扶持。全年新增亿元企业20家以上、规模工业企业100家以上。

构建现代产业体系。着力推进烟草、装备制造等六大主导产业转型升级，延伸产业链条，全年完成技改投入440亿元，增长12%。大力发展电子信息、生物医药、新材料、新能源等战略性新兴产业，年内新兴产业产值增长20%以上。推动信息化和工业化深度融合，抓好信息化在企业研发设计、生产管理、市场营销各环节的应用。积极创建名品名牌，新增中国驰名商标2件以上。

提升科技创新水平。强化企业在技术创新中的主体地位，建设一批国家和省级工程技术中心、重点实验室，抓好院士工作站、博士后工作站建设，让创新成为驱动发展的新引擎。继续实施博士创新创业行动，推进厅市合作、校企合作。组织一批重大科技专项，促进重点工艺、关键环节的技术突破。年内新增高新技术企业10家以上，高新技术产品产值达到650亿元。

支持中小微企业发展。加强财税扶持，整合安排资金，用于支持中小微企业发展。扩大信贷支持，引导各类金融机构、创业投资、股权投资投向中小微企业。优化涉企服务，加快推进市县中小企业综合服务平台建设，帮助搭建电商平台，在政府网站免费发布企业产品和市场需求信息。减轻企业负担，落实国家降费清税政策，公布涉企行政事业性收费和经营服务性收费清单，严禁清单之外的所有收费。在当前实体经济渡难关的关键时刻，各级政府要当好企业和企业家的坚强后盾，把解决企业的困难作为政府的工作重点，为企业发展搭上一把手、添上一把柴、加上一把火。

大力发展现代农业。坚持跳出农字抓农业、跳出农业抓农村的理念，推进农业的规模化、高端化、智能化、服务化，加快转变农业发展方式，提高农业综合生产能力。

提升主导产业。加快粮食、生猪、水产、蔬菜、林业五个“双百亿产业”发展，抓好柑橘、茶叶、油料、禽蛋等优势产业建设，积极发展高效特色农业产业。完善农业标准体系，加大标准示范和推广力度，建设农业标准化示范市。

推行现代经营。紧跟全省两个“百千万”工程步伐，大力扶持专业大户、农民合作社、家庭农场和农业经营企业的发展，全面推进新型职业农民培训工程，走新型主体快速发展之路。继续实施加工流通“双百双十工程”，大力发展农产品加工业和农业现代服务业，支持农业电子商务平台建设，走加工物流带动之路。积极推进土地承包经营权确权登记颁证工作，引导农村土地承包经营权规范有序流转，走适度规模经营之路。抓好“三品一标”认证工作，加大优质农产品品牌营销力度，走品牌带动之路。

完善服务体系。注重农业技术创新，加大优质种苗、先进实用技术推广力度，发展农作物病虫害专业化防治和绿色防控，进一步提高农业机械化综合水平。落实强农惠农政策，严格农民负担监管。积极推进农业保险。加强农业招商引资工作，鼓励引导工商资本、城市资本投入农业现代化。高度重视防汛抗旱工作。抓好森林防火、农产品质量安全和重大动植物疫病防控。

加强示范引领。全面推进国家农业科技园创建，加快西湖西洞庭国家现代农业示范区建设，支持每个区县(市)建设一个综合性现代农业示范区。按照“一乡一品、一村一业”的思路，抓好现代农业特色园区建设。

大力发展现代服务业。积极培育服务业发展新的增长点，努力打造区域消费中心。

促进旅游业加快发展。推进文化旅游深度融合，把文化旅游作为战略性新兴产业来培育。打响世外桃源旅游品牌，塑造“亲亲常德”旅游形象，加快建设国内外知名的休闲度假旅游目的地。以华侨城梦幻桃花岛水公园开园为契机，办好常德旅游节。完成世外桃源古镇主体工程，加快抗战英雄城、城头山、东山峰、壶瓶山、桃林花海、花山湿地、西洞庭湖湿地等项目的策划和建设，支持柳叶湖国家级旅游度假区和桃花源国际文化旅游度假区品牌创建。积极培育休闲游、度假游、体验游、乡村游等旅游业态，拓展旅游消费新热点。

促进金融业创新发展。大力推行PPP融资模式，积极运用TOT、BOT等融资方式，扩大有效信贷投入，全年新增银行贷款120亿元以上、表外融资120亿元以上。构建新型地方金融体系，加快组建农村商业银行、村镇银行和小额贷款公司，加大引进各类金融机构力度，积极支持在常商业银行网点下沉。鼓励金融产品创新，稳步推进土地承包经营权、农民住房财产权抵押贷款试点。加快企业上市步伐，实现益丰大药房在上交所上市。支持市城建投、市经建投等投融资公司做实做强，积极探索混合所有制改革在常德的具体运用。发展保险业，规范保险市场。更好发挥国有担保机构在支持中小微、“三农”等企业融资中的作用。推进社会信用体系建设，优化金融生态环境，加强民间融资风险管控，严厉打击恶意逃废金融债权和非法集资行为，维护金融稳定。

促进商贸物流业提质发展。加快城市商圈、大型专业市场、城市综合体建设，高标准完成武陵阁步行城商圈改造，加快推进友阿商城、湘西北汽贸城、农产品大市场等项目。完善市县两级物流网络，加快引进知名物流企业，鼓励本地物流企业做大做强。

促进互联网经济和电子商务跨越发展。实施“百千企业电商起航”转型升级工程，促进线上线下加快融合，建设武陵移动互联网产业园，开辟电商大楼，搭建创业平台，扶持本地电商企业发展壮大。

促进其它服务业稳步发展。培育壮大影视传媒、动漫游戏、新闻出版、演艺娱乐等文化产业。大力发展养老服务业、家政服务业和健康产业等生活服务业，拓展信息通讯等新兴服务消费。稳定住房消费，促进房地产业健康发展。全年完成社会消费品零售总额918亿元，增长12%。

规划建设柳叶湖现代服务业园区。年内启动建设市民服务中心、上市公司与投融资公司总部基地，规划建设金融街区。

大力发展县域经济。把县域经济作为全面建成小康社会的重要着力点，切实增强县域发展活力和整体实力。

推进经济提速。坚持分类指导，落实促进县域经济发展的政策措施，市财政支持县域经济的力度只增不减。推进扩权强县和扩权强镇，进一步下放管理权限，抓好石门县省直管县体制改革试点和陬市镇全国经济发达镇行政管理体制改革试点。鼓励发展县城经济、园区经济，壮大县域经济核心增长极。引导中心城镇发展特色产业，加快建设一批经济强镇。推进津市澧县全国中小城市综合改革试点工作，加快桃源全省特色县域经济重点县建设，支持有条件的县(市)积极申报特色经济重点县。提升市域干线交通网络，启动农村公路连通工程，实现县乡干线公路与国省干线公路有效连接。积极帮助县(市)争取上级支持，完善县域经济发展量化考核评价机制。

强化扶贫攻坚。继续实施“六件实事”扶贫攻坚三年行动，全面完成首轮160个贫困村脱贫任务，实现12万名扶贫对象稳定脱贫。实施“雨露计划”和农村实用人才培养计划，全年培训贫困农民5000人次以上。创新和完善精准扶贫、对口扶贫、社会扶贫机制。做好移民后扶工作。

重视基层工作。真情重视基层建设，真情关心基层干部，保障好他们的正常待遇。加快公共服务向农村延伸，加大乡村运转经费保障力度，改善乡村工作生活条件。尊重基层和群众的首创精神，大力培养一批优秀致富带头人，增强农村发展活力。

二、打牢发展根基，聚精会神搞建设

以更大的力度推进基础设施建设，更好地发挥投资对经济发展的关键作用。全年安排重点工程项目500个，年度投资1000亿元。

推进城镇建设。把握中央推进新型城镇化的要求，以人的城镇化为核心，加快打造泛湘西北现代化的区域中心城市，推进沅澧城镇群建设。完善城市规划体系。坚持“多规合一”，完成中心城区160平方公里控制性详规，做好城市产城融合、特色塑造、历史文化延续、江南江北统筹、三大水系风光带连通规划。增强城市承载功能。进一步完善中心城区路网体系，加快东片区、西片区道路建设，开工建设沅水四桥，做好沅江隧道建设前期工作，规划建设平战结合的地下停车场。抓好城镇雨污分流、穿紫河水系改造，完成棚户区改造3.6万户，抓好重点区域美化亮化工作，进一步完善智慧城管、智能交通、数字防控运行体系。继续推进柳叶湖、白马湖、智慧谷、江南新城、德山城区等重点片区建设。提升城市管理水平。完善“一改四化”体制机制，切实推进重心下移，严格考核考评。继续实施中心城区交通秩序和市容环境卫生两项整治，加强渣土运输、户外广告等管理，巩固提升城市创建成果和城市品质。

按照“一主一副四轴”发展框架，推进城乡统筹发展、市县互动发展。加快县(市)城区建设，完善市政基础设施和公共服务设施，推进县城“改镇设办”，增强要素集聚、产业集聚、人口集聚能力；提升小城镇发展水平，重视省际边界口子镇建设，推进产城融合，培育一批产业特色鲜明、基础设施完善、生活环境优美的现代新型城镇。全市城镇化率达到47%以上。

推进交通建设。坚持交通先行，积极谋划、加快构建区域立体化交通枢纽，尽快打造泛湘西北交通枢纽城市，从根本上提升常德的区位优势。加快黔张常铁路建设，启动常岳九、长益常、宜石常高铁前期工作；加快常安高速建设，启动和加快宜张高速、益常高速复线前期工作；加快沅水浦市至常德航道整治，完成德山港区千吨级码头一期主体工程，启动澧水千吨级航道建设；加快石龟山、陆家渡等桥梁建设，改造农村危桥及渡改桥129处；加快国道319和207市城区改线等项目建设，新建改建国省干线公路180公里。加强交通管理，保持公路治超高压态势，提高公路管养水平。

推进水利建设。加快实施中央和省投资工程，完成266处蓄洪垸堤防加固、85座水库除险加固，以及大中型灌区更新改造、中小河流治理等建设任务。推进沅澧两水堤防安全工程，抓好常德经开区防洪圈、鼎城区善卷垸等一线大堤建设。继续开展小型农田水利建设五年行动，加快解决农业灌溉“最后一公里”问题。实施农业综合开发和土地综合治理项目，建设高标准农田和改造中低产田15万亩。

推进能源建设。加快石门白沙渡水电站等项目建设。完善电网布局，启动实施酒泉至湖南特高压直流线路、常德北输变电工程、城乡中低压配电网等项目，继续实施农村电网改造。完成“五县一市”天然气输配管道工程，开工建设常德至西洞庭天然气长输管网。支持风力发电等项目开发。

推进美丽乡村建设。抓好示范引领，继续开展“百村示范、千村创建、村村整治”行动，重点推进100个美丽乡村示范村建设。突出环境整治，积极开展旧村改造，完善农村垃圾处理、面源污染防治、水环境治理和畜禽集中养殖污染治理措施。鼓励和引导农民积极建设美丽家园，广泛动员社会力量领建援建美丽乡村。继续开展“3+X”乡村治理模式试点。三、激发发展活力，积极稳妥抓改革

围绕解决发展面临的突出问题，稳步推进各项改革，使改革举措有效转化成发展动力。

全面深化改革。完成好中央和省里部署的各项改革任务。重点推进十项自主改革：深化市、区行政管理体制改革，界定市、区政府职能，理顺市、区两级政府关系；深化行政审批制度改革，进一步简政放权、扩权强县；深入推进完美社区建设，创新城市基层社会治理；深入推进美丽乡村建设，创新农村基层社会治理；深度融入洞庭湖生态经济区建设，探索以生态文明建设引领经济社会发展的新路径、新机制；深化产权制度和要素市场改革，完善市场体系和招投标平台，建立统一、透明、规范的公共资源交易市场；推进投融资体制改革，加快组建农村商业银行和村镇银行，防范债务风险；开展小城镇城管体制改革试点，选择10个小城镇赋予县城城市管理部分职能；统筹推进津澧融城，抓好国家中小城市综合改革试点；推进津市城乡一体化试点。

壮大民营经济。探索实行负面清单管理模式，放宽非公有制企业进入领域，鼓励发展非公有资本控股的混合所有制企业。依法保障非公有制企业合法权益。鼓励全民创业，制定和落实优惠政策、扶持措施，营造浓厚的社会氛围和宽松的营商环境，使创业创新成为人们普遍的生活方式，成为社会纵向流动的强大动力，促进大众创业、万众创新。

积极扩大开放。深化对外合作，加强与长江经济带、洞庭湖生态经济区和泛湘西北城市群的对接融合，加强与国内外友好城市、港澳台地区的联系交流。打造开放平台，积极申报公路口岸、铁路口岸、航空口岸，建成常德经开区公共保税仓，做大做强盐关水运口岸。扩大对外贸易，扶持出口企业开拓新兴市场、引进先进技术、境外上市融资，加快“走出去”步伐。抓好产业承接，重点引进一批龙头企业和战略项目；实施“德商回乡”战略，办好德商恳谈会等活动；组织央企恳谈会，加强与央企的对接。把招商引资作为“一把手”工程，主要领导、主要精力、主要资源要投入到项目引进中去，对招商引资决不能说起来重要、喊起来响亮、做起来挂空档，重大项目务必盯紧看牢、落地见效。全年引进内外资总额600亿元以上，其中直接利用外资6.2亿美元以上。

四、紧扣发展目的，尽心尽力惠民生

更加注重保障基本民生，深入打好民生升温战役，全面完成省为民办实事任务，让幸福常德成为我们的靓丽名片。

全面解决饮水安全问题。完成城乡饮水安全工程建设，解决好136万人的饮水安全问题。继续完善城乡饮水安全管理长效机制，切实加大水质检测和监测力度，提升城乡供水运行管理水平。全面完成饮用水源地划定与报批工作，落实严格的饮用水源地保护措施。

积极扩大就业。努力提升政府公共就业服务能力，切实承担好就业培训、就业援助的责任。突出抓好高校毕业生、城乡贫困家庭就业困难人员和退役军人等群体的就业帮扶。加强就业观念引导，积极帮助高校毕业生转变就业观念，增强创业就业意识。积极开展中长期技能培训，提高就业困难群体稳定就业技能。高度重视人才工作，在重点产业、急需领域培养和引进更多的优秀人才，全市高技能人才增加5000人、达到7万人。

完善社会保障。强化社保扩面主体责任，加大对“个私外”企业的扩面力度，年内五类社会保险新增参保12万人次以上。重视做好下岗失业人员、失地少地农民、灵活就业人员等群体的社会保障工作，积极帮助接续社会保险关系。做好养老保险制度改革工作。加快建立重特大疾病保障机制，进一步扩大单病种包干范围。提升城乡低保、农村五保、优抚对象等群体的救助水平，做好孤儿、重残智残、失独家庭等群体的救助工作。建设保障性安居工程5.4万户，全面实现廉租房与公租房并轨。加快社会化养老服务体系建设，办好一批示范养老机构。重视和发展慈善事业。

建设教育强市。深入推进教育三年攻坚，继续实施农村薄弱学校改造和城区中小学校扩容提质工程，新建北师大附属学校，建设一批社区公办幼儿园和普惠性民办幼儿园。进一步提升普通高中教育办学水平，抓好普职合理分流，提升职业教育质量，加快职教城建设。确保湖南幼师高专、常德技师学院搬进新区办学，积极申办常德财经职院，支持湖南文理学院申办硕士学位点。实施教育信息化工程。支持民办教育、特殊教育、老年教育发展。加强学校文化、师资力量、师德师风和平安校园建设。做好捐资助学工作。

抓好卫生计生工作。深入推进卫生三年攻坚，加快市县两级医疗服务中心建设，推进社区卫生计生服务中心新建和改扩建，加强基层医疗卫生人才培养和引进。完成县级公立医院综合改革。巩固完善基本药物制度，规范药品流通秩序。进一步提高新型农村合作医疗补助标准，稳定参合水平。做好疾病防控、妇幼保健、爱国卫生运动工作，加强医德医风建设。发展中医药事业，创建全国基层中医药工作先进市。坚持计划生育基本国策，加强依法管理，深化优质服务。

促进文化繁荣。培育和践行社会主义核心价值观，加强公民道德和精神文明建设。加快完善文化基础设施，推进乡村文化广场建设，创建国家公共文化服务体系示范区。广泛开展群众文化活动，鼓励和扶持文艺精品创作。推进文化惠民工程。重视文化遗产保护工作。加强文化市场监管和广电新闻出版管理。开展全民健身运动，提高竞技体育水平。

建设完美社区。按照“三年目标两年完成”的要求，确保今年硬件建设扫尾，社区公共服务用房全面达标，街道“一所一校四中心”、社区“一站一园一场五室”建设全面完成；充分运用信息化手段，促进服务机制改革，确保“3+N”社区治理模式全面推行，社区网格化管理全面落实。

发展各项事业。加强财税工作，抓好开源节流，开展政府购买服务，强化政府性债务风险防控。坚持节约集约用地，做好征地拆迁安置工作，保障重大项目用地需求。加强知识产权保护和运用，推广普及科技知识。抓好住房公积金归集和资金管理，提高资金使用效益。发展老龄事业，做好老干部工作。完善残疾人保障服务体系，落实好扶残助残的各项政策。支持革命老区加快发展。做好审计、外事侨务、统计、民族宗教、防震减灾、方志、档案、保密、对台、气象、驻外机构等工作。支持工会工作，切实保障职工群众的权益。重视支持共青团和妇女儿童工作。

加强新形势下国防动员、国防教育、人民防空和兵役工作，统筹抓好经济发展与国防后备力量建设，完善军民融合深度发展机制，深入推进“双带双促”和“双拥”共建活动，争创全国“双拥”模范城。

五、守住发展底线，千方百计保安全

留住青山绿水，维护平安稳定，是我们必须坚守的底线，也是义不容辞的职责。

守住社会稳定底线。大力开展各类平安创建行动，创新社会治理方式，健全立体化的社会治安防控体系，严密防范和依法惩治各类违法犯罪活动，强力推进禁毒工作社会化。落实重大决策社会稳定风险评估机制，把维稳的关口前移，防止侵害群众利益的现象发生。大力推行阳光信访，坚持领导干部下访和部门联合接访，畅通信访渠道，落实信访积案化解工作责任，依法处理信访活动中的违法犯罪行为。重视网络舆情监督，依法加强网络社会管理。完成“六五”普法验收，加强人民调解、法律援助和社区矫正工作。

守住公共安全底线。严格落实安全生产责任制，持续深入开展打非治违、隐患排查和专项整治行动，坚决防止重特大安全事故发生。抓好食品药品监管体制改革，夯实监管基层基础，强化重点品种和热点问题专项治理，创建一批食品药品安全示范单位。完善各类公共事件应急预案，建立建强应急救援力量，加强应急演练。

守住生态环保底线。实施蓝天碧水净土行动，推进大气污染防治，严格落实工业污染减排、淘汰落后产能、“煤改气”、机动车环保管理、渣土车辆运输监控、建筑工地扬尘监管等措施，确保空气质量稳定上升，市城区空气质量达标率力争达到90%以上。扎实推进水环境保护和水生态建设，在河流、湖泊、水库、公共水域全面禁止投肥养殖，在沿河、沿湖区域全面开展养殖污染治理行动，在农村“五小”水利建设中全面推行生态护坡，增强水体自我净化功能。抓好生态文明示范区和全国土壤污染综合防治示范区建设，推进污染减排体系建设，启动工业企业排污权有偿使用和交易工作。加强森林、湿地生态修复，积极创建国家森林城市。努力让蓝天常在、青山常在、绿水常在。

科学编制“十三五”规划是今年的一项重大任务。要从常德实际出发，深入研究经济新常态的发展规律，准确把握面临的重大机遇和挑战，科学提出今后五年的发展目标和任务，使规划成为凝聚力量和指引发展的项目库、路线图、任务书。

各位代表!

新形势新任务对政府自身建设提出了更高的要求。各级政府和政府工作人员要自觉践行“三严三实”，坚决守法治，坚定守承诺，坚持守廉洁，不负全市人民的信任和重托。在政府工作中，我们要始终坚持的是：

以扎实的举措践行法治。牢固树立法治思维，努力建设法治政府、法治社会、法治常德。做到依法决策。对重大决策事项，严格落实公众参与、专家论证、风险评估、合法性审查、集体讨论决定的程序，建立重大决策终身责任追究制度和责任倒查机制。关系群众切身利益的重大事项，在决策前都要实行公示听证。做到依法行政。严格按照法定权限和程序行使权力、履行职责。全面落实行政执法责任制，健全行政裁量权基准制度，坚决纠正不作为、乱作为和执法不公、违规执法的问题。做到依法办事。严格办事服务程序，提高运用法治思维和法治方式深化改革、推动发展、化解矛盾、维护稳定的能力，形成办事依法、遇事找法、解决问题用法、化解矛盾靠法的良好法治氛围。

以优良的作风提升服务。努力为企业、基层和群众提供优质高效服务，重点抓好五件事：一是机构改革。全面完成新一轮政府职能转变和机构改革工作，进一步调整完善机构，理顺职能职责，改进管理方式，提高行政效能，建立健全行为规范、运转协调、公正透明、廉洁高效的行政管理体制。二是简政放权。规范行政审批管理，制定和公布“四张”清单，即市场准入负面清单、政府权力清单、责任清单和各项政策清单。三是效能提速。提升政务中心、社区服务中心、社会求助服务中心、政府门户网站等平台建设水平，积极推行网上办事，全面落实全程代理、并联审批、超时默认等制度。强化政府执行力，确保市委作出的决策部署、市政府抓的各项工作、对群众的各项承诺，定一件办一件，办一件成一件。四是一线解难。市政府领导和政府部门分别联系一家重点企业、一个重大项目、一个村或社区、一所学校或乡镇卫生院，深入开展调研，实施结对帮扶，帮助解决困难问题。五是优化环境。认真清理和规范中介机构、行业协会，下大力治理“三乱”，严厉整治扰乱经济秩序的违法行为，严厉整治招投标中的暗箱操作、串标围标、层层转包等违法行为，严厉整治建设过程中的强行阻工、强揽工程、强买强卖等违法行为，营造公平竞争的市场环境、安宁和谐的建设环境、重商亲商的人文环境。

以诚恳的态度接受监督。政府的一切权力来源于人民。我们一定主动接受人民监督，努力建设阳光政府。自觉接受市人大及其常委会的法律监督和工作监督，执行市人大及其常委会的决议决定，落实好规范性文件备案审查和“三统一”制度。支持和保障市政协履行政治协商、民主监督、参政议政职能，认真办理人大代表建议和政协提案，完善社情民意办理反馈机制，虚心听取和采纳各民主党派、工商联、无党派人士的意见建议，接受新闻舆论和社会公众监督，加强监察、审计等内部监督。加快推进政务公开，全面推行行政权力运行、财政资金、公共资源配置、公共服务和公共监管等各类信息公开，加强电子政务建设，方便群众办事和监督。

以严明的纪律守住廉洁。坚持从严治政，努力做到干部清正、政府清廉、政治清明。严格遵守改进作风的各项规定，坚决反对铺张浪费和大手大脚，自觉抵制享乐主义和奢靡之风。今年，“三公”经费继续从严从紧、只减不增。严格落实党风廉政建设责任制，严格执行廉洁从政各项规定，严格查处各类违法违纪案件，以实际行动取信于民。

各位代表!光荣和梦想召唤着我们，责任和使命激励着我们。让我们在市委的坚强领导下，凝心聚力，开拓创新，奋发进取，为加快推进新常德新创业的宏伟事业而努力奋斗!
